# A Cross-Sectional Analysis of Health Behavior in School-Aged Children: The Qassim Study

**DOI:** 10.7759/cureus.48420

**Published:** 2023-11-07

**Authors:** Abdelmarouf Mohieldein, Mahmoud Elhabiby, Ayman Abu Mustafa, Modather Shehade, Sultan Alsuhaibani

**Affiliations:** 1 Department of Medical Laboratories, College of Applied Medical Sciences, Qassim University, Buraidah, SAU; 2 Department of Medical Laboratory Sciences, Al-Aqsa University, Gaza, PSE; 3 College of Nursing, Ministry of Health, Gaza, PSE; 4 Department of Medical Laboratories, Almustaqbal University, Buraidah, SAU

**Keywords:** school-aged children, saudi arabia, childhood obesity, physical activity, dietary habits

## Abstract

Background

Childhood obesity is a worldwide public health epidemic. Sedentary lifestyles and unhealthy dietary patterns increased the rates of overweight and obesity among children. This study aimed to (1) estimate healthy behaviors (including dietary patterns and physical activity) and (2) determine the prevalence of overweight and obesity among Saudi school-aged children in the Qassim region, Saudi Arabia.

Methods

A population-based cross-sectional study was conducted among Saudi schoolchildren aged between five and 16 years old. The study group consisted of 339 children including 237 males and 102 females. Data were collected using a questionnaire based on the Health Behavior in School-Aged Children (HBSC) survey. Body mass index (BMI) for age and gender was computed for each child using the AnthroPlus 2007 software (World Health Organization {WHO}, Geneva, Switzerland). Statistical Package for Social Sciences (SPSS) version 23.0 (IBM SPSS Statistics, Armonk, NY) was used for data analyses.

Results

The prevalence of overweight and obesity among Saudi children was 46 (13.6%) and 81 (23.9%), respectively. Males consumed more soft drinks and energy drinks, whereas females consumed more fruit juice. As children grew older, the consumption of unhealthy food and beverages increased. Males engaged in more daily physical activity compared to females.

Conclusion

Saudi schoolchildren (aged 5-16 years) demonstrated significant gender-specific variations in dietary patterns and levels of physical activity. A significant proportion of Saudi children were overweight or obese. The study highlighted the complex relationship between age, school class, gender, and health behaviors among Saudi school-aged children. Policymakers and parents could benefit from the understanding of such complex relationships to promote healthy behaviors among school-aged children.

## Introduction

Childhood obesity is a global public health epidemic. It has an increased prevalence worldwide including developed and low- and middle-income countries, as well as countries of the Arabian Peninsula [1,2]. Childhood obesity has been described by the World Health Organization (WHO) as "one of the most serious public health challenges of the 21st century" [3]. According to the 2016 WHO reports, one out of five children and adolescents, aged 5-19 years, were found to be overweight or obese [4]. In the Arab Gulf countries, childhood obesity has reached an alarming level [5]. The dietary pattern toward westernized fast foods, unhealthy snacks, and soft drinks in Saudi Arabia has become common among the population due to the significant socioeconomic growth during the recent decades [6,7].

Parents usually face many challenges in encouraging and establishing healthy dietary habits among their children as a result of demands driven by contemporary society [8]. For the health and well-being of the children, it is necessary to establish healthy eating habits, which have an influence on the physical, emotional, and psychological development of the child [9]. Hence, such a shift in dietary patterns and lifestyles results in a change in deaths caused by diet-related non-communicable diseases instead of communicable diseases. These diet-related diseases include obesity, cardiovascular diseases, and type 2 diabetes mellitus [10]. To decrease the risk of serious diseases such as obesity, it is important to have balanced and healthy food, as well as regular physical activity, both of which will help children's survival and well-being [11]. The 2010 US Department of Agriculture (USDA) Dietary Guidelines recommended decreasing the consumption of calorie-dense foods such as sweets and protein sources with highly saturated fats while increasing the eating of fruits, vegetables, and whole grains [12].

Childhood is the most critical stage to establish healthy behaviors such as having balanced and healthy food and increased physical activity. These healthy behaviors can affect not only the child's health but also its future health. It is obvious that children with unhealthy dietary habits will continue to maintain the same habits as they grow older [13,14]. Healthy eating is the eating practice and behavior that are consistent with improving, maintaining, and/or enhancing health. Hence, unhealthy eating habits can hinder optimal growth and development during childhood [15]. According to the WHO, a healthy diet is characterized by a balance between an energy expenditure and an energy intake. It should contain nutritious components such as fruits, vegetables, legumes, nuts, and whole grains, with limited amounts of free sugars, salt, and fat [16]. Several interdependent factors can influence the dietary habits of the children. However, a child's home environment has a significant influence on the establishment of healthy behaviors that will persist throughout a child's life [17]. Therefore, a better understanding of the current dietary habits of the children will help in the establishment of new programs to manage childhood obesity, which is one of the diet-related non-communicable diseases [18]. Around 30%-35% of the total Saudi population are children under 16 years old [19]. We aimed to (1) estimate the daily dietary habits and physical activity and (2) determine the prevalence of overweight and obesity among Saudi children aged between five years and 16 years in the Qassim region, Saudi Arabia. There is a lack of comparable data on dietary habits and physical activity among school-aged children in the Qassim region, Saudi Arabia.

## Materials and methods

Study design and population

A population-based cross-sectional study was conducted among Saudi schoolchildren aged between five and 16 years old. The study group consisted of 339 children, of whom 237 were males and 102 were females. The inclusion criteria were Saudi citizens, both male and female, enrolled in primary or intermediate schools, and an age range of five to 16. The exclusion criteria were non-Saudi citizens, older than sixteen, and preschool-aged children.

Saudi Arabia's general education system consists of primary school (grades 1-6), intermediate school (grades 7-9), and secondary school (grades 10-12). The children in our study were selected from those who attended the Buraidah Spring Festival.

Data collection

We collected data using a questionnaire based on the Health Behavior in School-Aged Children (HBSC) survey. The HBSC survey has been found to have reliable validity in relation to 24-hour and seven-day food diary tools [14]. To ensure the questionnaire's validity, accuracy, and clarity, we initially translated the questionnaire into Arabic; then, we conducted pretesting. Well-trained research assistants administered the questionnaires. The research assistants received training prior to data collection with special emphasis on standardizing the methods of measurement. The children and parents were informed about the study and the potential benefits of participation. The benefits included gaining insights into the optimal body mass index (BMI) for children based on their age and gender, as well as the understanding of the negative effects of consuming unhealthy food and beverages. For children who could not fill out the questionnaire, the research assistants read for them and allowed the child to select his or her choices.

Dietary and physical activity assessment

The questionnaire included a series of food and beverage items so as to provide an indication of the quality of diet among school-aged children. Children were asked, "During last week, how often did you consume each of the following?" The response options were the following: never, once a week, two to four days a week, five to six days a week, once a day, or more.

The dietary items were categorized into two groups: (A) healthy food and beverage items that include fruit juice, whole-fat milk, cheese, cereals, and brown bread and (B) unhealthy food and beverage items that include soft drinks, energy drinks, French fries (chips), potato chips (crisps), and sweets.

Fat intake was evaluated by analyzing the consumption of chips and crisp items, while sugar intake was evaluated by examining the consumption of white bread, soft drinks, energy drinks, and sweets items. Calcium intake was assessed based on the consumption of whole-fat milk and cheese items, while the intake of vitamins and trace elements was evaluated in relation to the consumption of fruit juice, cereals, and brown bread.

The term "physical activity" refers to any body movement that involves the contraction of skeletal muscles and the use of energy [20]. Physical activity for the children in the study was estimated by asking about the frequency of spending 30 minutes per day on various physical activities during the previous week.

Anthropometric measurements

The height and weight were measured for each child using the appropriate instrument according to a standard protocol. BMI for age and gender was computed for each child using the WHO software AnthroPlus 2007 (Geneva, Switzerland). This program deduced z-score and percentiles using the exact age in days. BMI status was interpreted, based on the norm for age and sex, and children were classified into four groups: underweight (if ≤15th percentile), normal weight (16th-84th percentile), overweight or at risk for obesity (85th-94th percentile), and obese (≥95th percentile) [21].

Statistical analysis

Statistical Package for Social Sciences (SPSS) version 23.0 (IBM SPSS Statistics, Armonk, NY) was used for data management and analyses. Descriptive statistics was employed: number (percentage) for categorical variables and mean ± standard deviation for continuous variables. A comparison between males and females was done by unpaired t test for continuous data while the chi-square test for categorical data. A correlation between continuous variables and variables related to dietary habits and physical activity levels was measured by applying the Pearson correlation coefficient (r). P < 0.05 was considered statistically significant.

Ethical considerations

The present study was approved by the ethical committee of the Department of Medical Laboratories, College of Applied Medical Sciences, Qassim University. Verbal informed consent was requested and obtained from children's parents after a thorough explanation of the study's goal, which was to provide insight into the optimal BMI for children based on their age and gender, as well as the negative impacts of consuming unhealthy food and beverages. The study was conducted as a part of the initiatives undertaken by the Department of Medical Laboratories in collaboration with the Deanship for Community Service at Qassim University during the Buraidah Spring Festival in the Qassim region, Saudi Arabia. Participation in the study was voluntary, and the confidentiality of the children was maintained as no names were requested in the questionnaires.

## Results

Sociodemographic data among school-aged children

A total of 339 Saudi schoolchildren, aged 5-16 years, participated in the current study. The majority of the children were in the 12-14-year-old age group, which represented approximately half of the participants. Males were predominated and constituted nearly two-thirds of the participants. According to Saudi Arabia's general education system, approximately two-thirds of the children were attending primary school. The prevalence of overweight and obesity in the children was 127 (38%), while half of the children (170, 50.1%) had a normal weight (16th-84th percentile). Table 1 presents the sociodemographic data of the study children.

**Table 1:** Sociodemographic data among the school-aged children Underweight or at risk for underweight, less than or equal to the 15th percentile; normal weight, 16th-84th percentile; overweight or at risk for obesity, 85th-94th percentile; and obese, more than or equal to the 95th percentile N, number of children; SD, standard deviation; BMI, body mass index

Sociodemographic data		N	%	Mean ± SD
Age of child (years)		-		10.99 ± 2.29
Age groups	5-8 years	59	17.4%	-
	9-11years	126	37.2%	-
	12-14 years	142	41.9%	-
	15-16 years	12	3.5%	-
Gender	Male	237	69.9%	-
	Female	102	30.1%	-
School level	Primary	234	69.0%	-
	Intermediate	105	31.0%	-
Weight (kg)	-	-	-	38.24 ± 13.26
Height (cm)	-	-	-	138.51 ± 12.81
BMI	-	-	-	19.43 ± 4.77
BMI status groups	Underweight	42	12.4%	-
	Normal weight	170	50.1%	-
	Overweight	46	13.6%	-
	Obese	81	23.9%	-

Dietary habits and physical activity levels among the school-aged children

Table 2 illustrates the dietary habits and physical activity levels of the school-aged children. Data analysis revealed that soft drinks were frequently consumed by the children, and only 54 (15.9%) of the children declared that they never intake soft drinks. On the contrary, the majority of Saudi children rarely consumed energy drinks (315, 92.9%). Fruit juice intake was moderate, with 95 (28.0%) of the children reporting daily consumption. Moreover, the study highlighted that white bread was the preferred choice among children, with most of the children consuming white bread daily. However, nearly three-quarters of the children reported that they never intake brown bread. In addition, sweets, crisps, and chips were consumed relatively frequently by the Saudi children. Concerning dairy, whole-fat milk was highly consumed, while skimmed milk intake was uncommon. Other milk products and cheese were also often consumed. In regard to physical activity, the children participated in moderate levels of physical activity, with an average of 5.32 times per week.

**Table 2:** Dietary habits and physical activity levels among school-aged children N, number of children; SD, standard deviation

Consumption frequency of items	Times/week					
	N (%)					Mean ± SD
	Never	1	2-4	5-6	7+	
Soft drinks	54 (15.9%)	75 (22.1%)	125 (36.9%)	23 (6.8%)	62 (18.3%)	2.98 ± 0.14
Fruit juice	40 (11.8%)	70 (20.6%)	99 (29.2%)	35 (10.4%)	95 (28%)	3.61 ± 0.17
Energy drinks	315 (92.9%)	12 (3.5%)	06 (1.8%)	03 (0.9%)	03 (0.9%)	0.2 ± 0.04
Skimmed milk	273 (80.5%)	06 (1.8%)	14 (4.1%)	25 (7.4%)	21 (6.2%)	0.98 ± 0.15
Whole-fat milk	64 (18.9%)	32 (9.4%)	63 (18.6%)	28 (8.3%)	152 (44.8%)	4.24 ± 0.18
Cheese	24 (7.1%)	18 (5.3%)	75 (22.1%)	29 (8.6%)	193 (56.9%)	5.17 ± 0.15
Other milk products	37 (10.9%)	42 (12.4%)	93 (27.4%)	29 (8.6%)	138 (40.7%)	4.27 ± 0.17
Cereals	161 (47.5%)	52 (15.3%)	54 (15.9%)	17 (5.1%)	55 (16.2%)	2.04 ± 0.17
White bread	18 (5.3%)	5 (1.5%)	15 (4.4%)	18 (5.3%)	283 (83.5%)	6.28 ± 0.12
Brown bread	248 (73.2%)	21 (6.2%)	13 (3.8%)	26 (7.7%)	31 (9.1%)	1.24 ± 0.16
Sweets	19 (5.6%)	47 (13.9%)	106 (31.3%)	38 (11.1%)	129 (38.1%)	4.36 ± 0.16
Crisps	63 (18.6%)	50 (14.7%)	102 (30.1%)	31 (9.2%)	93 (27.4%)	3.47 ± 0.18
Chips	23 (6.8%)	38 (11.2%)	112 (33%)	33 (9.8%)	133 (39.2%)	4.38 ± 0.16
Physical exercise of 30 minutes/day	27 (8%)	28 (8.3%)	43 (12.7%)	28 (8.2%)	213 (62.8%)	5.32 ± 0.16

Relationship between gender and dietary habits and levels of physical activity in school-aged children

Findings in Table 3 reveal gender-specific variations in the dietary habits and physical activity levels among Saudi school-aged children. Regarding beverages, males significantly consumed more soft drinks than females (3.39 ± 0.15 versus 2.02 ± 0.22 times/week, respectively; P = 0.000). Likewise, males had a higher weekly intake of energy drinks compared to females (0.27 ± 0.07 versus 0.03 ± 0.03 times/week, respectively; P = 0.027). However, females apparently consumed more fruit juice compared to males (4.03 ± 0.25 versus 3.43 ± 0.17 times/week, respectively; P = 0.051). Regarding the food items, there were no significant gender differences in the consumption of white bread, sweets, crisps, or chip. However, females significantly consumed more cereals than males (2.75 ± 0.28 versus 1.74 ± 0.16 times/week, respectively; P = 0.001). Data on physical activity engagement showed that males were significantly active in physical exercise when compared to females (5.64 ± 0.15 versus 4.56 ± 0.27 times/week, respectively; P = 0.000).

**Table 3:** Gender differences in dietary habits and physical activity levels among school-aged children *Significant at P < 0.05 n, number of children; SD, standard deviation; t, independent t test

Consumption frequency of items and physical activity levels	Gender (times/week), mean ± SD		Statistical test	
	Males (n = 237)	Females (n = 102)	t	P-value
Soft drinks	3.39 ± 0.15	2.02 ± 0.22	5.042	0.000*
Fruit juice	3.43 ± 0.17	4.03 ± 0.25	-1.961	0.051
Energy drinks	0.27 ± 0.07	0.03 ± 0.03	2.225	0.027*
Skimmed milk	0.99 ± 0.14	0.96 ± 0.21	0.111	0.911
Whole-fat milk	4.32 ± 0.19	4.07 ± 0.29	0.741	0.459
Cheese	5.13 ± 0.16	5.26 ± 0.22	-0.462	0.644
Other milk products	4.38 ± 0.17	4.01 ± 0.27	1.157	0.248
Cereals	1.74 ± 0.16	2.75 ± 0.28	-3.299	0.001*
White bread	6.32 ± 0.12	6.2 ± 0.19	0.534	0.594
Brown bread	1.34 ± 0.16	1.01 ± 0.22	1.161	0.247
Sweets	4.2 ± 0.16	4.73 ± 0.24	-1.844	0.066
Crisps	3.35 ± 0.17	3.75 ± 0.27	-1.262	0.208
Chips	4.35 ± 0.16	4.47 ± 0.23	-0.420	0.675
Physical exercise of 30 minutes	5.64 ± 0.15	4.56 ± 0.27	3.693	0.000*

Relation between school class and dietary habits and physical activity levels among the school-aged children

Table 4 reveals the significant variations in dietary preferences among primary and intermediate school students. Primary school pupils exhibited a higher average weekly consumption of fruit juice compared to their intermediate school counterparts (3.96 ± 0.17 versus 2.85 ± 0.24 times/week, respectively; P = 0.000). They also had significantly higher mean intakes of whole-fat milk and cereals compared to intermediate school students. On the other hand, intermediate school students demonstrated significantly higher mean intakes of soft drinks, energy drinks, and crisps per week when compared to primary school pupils. There were no significant differences in the mean consumption of skimmed milk, cheese, other milk products, white bread, brown bread, sweets, chips, or daily physical activity of 30 minutes between primary and intermediate school students (P > 0.05).

**Table 4:** The relationship between school class and the dietary habits and physical activity levels of the school-aged children *Significant at P < 0.05 n, number of children; SD, standard deviation; t, independent t test

Consumption frequency of items and physical activity levels	School class (times/week), mean ± SD		Statistical test	
	Primary (n = 234)	Intermediate (n = 105)	t	P-value
Soft drinks	2.5 ± 0.14	4.05 ± 0.24	-5.776	0.000*
Fruit juice	3.96 ± 0.17	2.85 ± 0.24	3.722	0.000*
Energy drinks	0.11 ± 0.04	0.4 ± 0.13	-2.633	0.009*
Skimmed milk	1.13 ± 0.15	0.64 ± 0.17	1.956	0.051
Whole-fat milk	4.47 ± 0.18	3.74 ± 0.29	2.168	0.031*
Cheese	5.21 ± 0.15	5.09 ± 0.25	0.445	0.657
Other milk products	4.41 ± 0.17	3.95 ± 0.26	1.498	0.135
Cereals	2.32 ± 0.17	1.42 ± 0.24	2.962	0.003*
White bread	6.27 ± 0.12	6.3 ± 0.19	-0.143	0.886
Brown bread	1.32 ± 0.16	1.07 ± 0.23	0.890	0.374
Sweets	4.42 ± 0.16	4.21 ± 0.24	0.716	0.474
Crisps	3.28 ± 0.18	3.9 ± 0.25	-1.994	0.047*
Chips	4.41 ± 0.16	4.34 ± 0.25	0.235	0.815
Physical exercise of 30 minutes	5.32 ± 0.16	5.3 ± 0.25	0.100	0.920

Correlation between age/school class/BMI and dietary habits and physical activity levels

Table 5 displays the findings of the Pearson correlation analysis between numerical variable (age, BMI, and school class) and items for dietary habits and physical activity levels. The results highlight positive correlations between age and soft drink intake (r = 0.297; P = 0.000), as well as energy drink intake (r = 0.137; P = 0.012). Conversely, age showed inverse correlations with fruit juice intake (r = -0.165; P = 0.002), whole-fat milk intake (r = -0.162; P = 0.003), and the consumption of sweets (r = -0.123; P = 0.023).

**Table 5:** Correlation between numerical data with dietary habits and physical activity levels among school-aged children *Statistically significant difference at P < 0.05 r, Pearson correlation coefficient; BMI, body mass index

Dietary habits and physical activity levels	Age (years)		School class (years)		BMI (child)		Physical exercise (30 minutes)	
	r	P-value	r	P-value	r	P-value	r	P-value
Soft drinks	0.297	0.000*	0.317	0.000*	0.158	0.004*	0.097	0.076
Fruit juice	-0.165	0.002*	-0.211	0.000*	-0.054	0.324	0.264	0.000*
Energy drinks	0.137	0.012*	0.123	0.024*	0.117	0.032*	0.034	0.536
Skimmed milk	-0.077	0.159	-0.113	0.037*	-0.008	0.879	0.104	0.057
Whole-fat milk	-0.162	0.003*	-0.153	0.005*	-0.079	0.146	0.089	0.101
Cheese	-0.037	0.501	-0.064	0.241	-0.067	0.216	0.076	0.161
Other milk products	-0.054	0.325	-0.062	0.256	-0.001	0.988	0.173	0.001*
Cereals	-0.168	0.002*	-0.195	0.000*	-0.065	0.236	0.085	0.119
White bread	-0.014	0.798	0.016	0.774	-0.026	0.629	-0.058	0.286
Brown bread	-0.012	0.820	-0.070	0.197	-0.027	0.620	0.105	0.052
Sweets	-0.123	0.023*	-0.111	0.041*	-0.109	0.046*	0.010	0.849
Crisps	0.073	0.177	0.087	0.112	0.021	0.702	0.131	0.016*
Chips	-0.084	0.122	-0.068	0.213	-0.101	0.063	0.118	0.030*
Physical exercise of 30 minutes	-0.020	0.709	0.005	0.922	-0.179	0.001*	-	-

Additionally, the school class exhibited positive correlations with soft drink intake (r = 0.317; P < 0.001) and energy drink intake (r = 0.123; P = 0.024) but negative correlations with fruit juice intake (r = -0.211; P < 0.001), skimmed milk intake (r = -0.113; P = 0.037), whole-fat milk intake (r = -0.153; P = 0.005), cereals (r = -0.195; P < 0.001), and sweets (r = -0.111; P = 0.041).

Interestingly, BMI demonstrated a significant positive correlation with soft drink intake (r = 0.158; P = 0.004) and energy drink intake (r = 0.117; P = 0.032) but a negative correlation with engagement in daily physical activity (r = -0.179; P = 0.001).

## Discussion

Sweetened beverages and calorie-rich snacks are available everywhere for children. They can be found in retail stores, school snack counters, and vending machines. This widespread availability of unhealthy dietary items may play a role in the increasing prevalence of childhood obesity worldwide [22].

Data from this study revealed that the prevalence of overweight (85th-94th percentile) and obesity (≥95th percentile) among Saudi children (aged 5-16 years) in the Qassim region was 46 (13.6%) and 81 (23.9%), respectively. Consistent with our findings, published reports from different regions of Saudi Arabia showed that the prevalence of overweight and obesity among Saudi children ranged from 10.6% to 23.1% and 13.5% to 30.4%, respectively [23-27]. The variation in the reported prevalence rates of overweight and obesity among Saudi children across different regions in Saudi Arabia highlights the demand for exploring childhood obesity. Childhood obesity can have both health and economic implications. In Arab countries, the reported prevalence of overweight and obesity in Kuwait was 25.5% and 36.5%, respectively [28]; in the United Arab Emirates, it was 21.5% and 13.7% [29]. Moreover, 18.5% of the children in the USA were reported to be obese, while 7% of the children around the world were estimated to be obese [30].

It is necessary to increase children's awareness toward healthy choices since they are the target for such widespread and affordable unhealthy food and beverages. Therefore, the WHO has called for effective strategies to address the growing epidemic of childhood obesity, which has been aimed to be stopped by 2025 [31]. Rapid urbanization and civilization have resulted in a change in dietary patterns toward unhealthy dietary habits that are associated with diminished levels of physical activity. Accordingly, the inevitable outcome is a global increase in the prevalence rates of overweight and obesity [32]. Hence, we are interested in getting a comprehensive understanding of the dietary habits and the level of physical activity among the Saudi school-aged children in the Qassim region, Saudi Arabia. We applied an HBSC-based survey so as to get new insights about the health behaviors including dietary patterns and the level of physical activity among these Saudi school-aged d children.

To develop healthy behaviors, it is necessary for a child to maintain a balanced diet with optimum physical activity [33]. Childhood is a crucial stage in the human development. Establishing healthy behaviors during childhood could have an effective impact during adulthood. Therefore, the adoption of healthy dietary choices and healthy lifestyle practices in childhood deserves significant attention [34]. As presented in Table 2, there was a preference among Saudi school-aged children for the consumption of soft drinks, white bread, whole-fat milk, other milk products, cheese, sweets, crisps, and chips. Moreover, findings in Table 3 revealed gender-specific variations in daily dietary patterns and physical activity levels among Saudi school-aged children. Males significantly consumed more soft drinks and energy drinks compared to females. Nevertheless, females apparently consumed more fruit juice than males. These findings are consistent with an Indian cross-sectional study that reported that female schoolchildren consumed fruits more than their male counterparts [35].

The consumption of sugar-sweetened beverages has negative health effects such as obesity and type 2 diabetes [36]. As reported in Table 5, Saudi school-aged children showed a decline in dietary quality as they grew older. Particularly, there were positive correlations between age and soft drink intake (r = 0.297; P = 0.000) and energy drink intake (r = 0.137; P = 0.012) but inverse correlations with fruit juice intake (r = -0.165; P = 0.002), whole-fat milk intake (r = -0.162; P = 0.003), and the consumption of sweets (r = -0.123; P = 0.023). Interestingly, BMI demonstrated a significant positive correlation with soft drink intake (r = 0.158; P = 0.004) and energy drink intake (r = 0.117; P = 0.032) but a negative correlation with the level of daily physical activity (r = -0.179; P = 0.001). These findings highlight the urgent need for the implementation of healthy behaviors among Saudi school-aged children across various age groups.

Based on data reported by the French Food Safety Agency, French children (aged 3-14 years) have shown an 11% decline in the consumption of dairy products [37]. Recently, it has been reported that 40% of American children (aged 2-18 years) meet the recommended intake of fruits and vegetables. The consumption of fruits and vegetables was inversely correlated with age [38]. The consumption of sugary beverages represents a global health challenge [39]. Ultra-processed foods include items such as soft drinks, sweets, snacks, chocolate, cookies, pastries, cereals, nuggets, sausages, burgers, hot dogs, "instant" soups, noodles, and desserts. These products are considered to have a poor nutritional value [40]. The Dietary Guidelines for Americans recommend the consumption of a variety of nutrient-rich foods. These healthy foods are categorized into five groups: vegetables, fruits, whole grains, low-fat or nonfat dairy products, and high-quality protein sources. Moreover, it is advisable to replace sweetened beverages with water, milk, or fruit-based drinks in schools [41]. As documented, both unhealthy dietary choices and a sedentary lifestyle lead to obesity and ultimately to death [42]. Regular physical activity has a positive influence on a child's mental and physical health. Consequently, promoting such cognitive capabilities and emotional stability could contribute to enhancing the academic performance of school-aged children [43]. Data analysis from the current study indicated that Saudi school-aged children were engaged in moderate levels of physical activity. However, males were significantly more active in physical exercise compared to females (5.64 ± 0.15 versus 4.56 ± 0.27 times/week, respectively; P = 0.000). A study reported that a recognized proportion of Kuwaiti children did not engage in daily physical activities. Specifically, 44.4% of males and 76.0% of females did not meet the global recommendations for daily physical exercise [44].

Limitations

This study has two limitations that could be addressed in future investigations. First, we did not calculate the total calorie content of food and beverage items. Instead, we asked about the frequency of intake during a previous one-week duration. Assessing the total calorie intake can provide a comprehensive understanding of calorie overconsumption, which is the main cause of childhood obesity. Second, we applied a convenience sampling method rather than random selection; i.e., the children who were invited were attendees of the Buraidah Spring Festival. This convenience sampling method could limit the generalization of the study's outcomes.

## Conclusions

In conclusion, Saudi schoolchildren (aged 5-16 years) demonstrated significant gender-specific variations in dietary patterns and levels of physical activity. A significant proportion of Saudi children were overweight or obese. The study highlighted the complex relationship between age, school class, gender, and health behaviors among Saudi school-aged children. Policymakers and parents could benefit from the understanding of such complex relationships to promote healthy behaviors among school-age children.
